# Effects of seedling age and cultivation density on agronomic characteristics and grain yield of mechanically transplanted rice

**DOI:** 10.1038/s41598-017-14672-7

**Published:** 2017-10-26

**Authors:** Qihua Liu, Xuebiao Zhou, Jingling Li, Caiyun Xin

**Affiliations:** Shandong Rice Research Institute, Shandong Academy of Agricultural Sciences, Jinan, 250100 China

## Abstract

Delayed transplantation frequently occurs in mechanically transplanted rice in China, leading to a significant reduction in grain yield. Thus, determining how to compensate grain yield loss is crucial for improving rice cultivation technology. A field experiment was conducted to investigate the effects of cultivation density and seedling age on agronomic traits and grain yield of mechanically transplanted rice. With increasing seedling age, rice tiller number, pre-anthesis dry matter accumulation, remobilization efficiency and contribution to grain yield, as well as post-anthesis photosynthesis amount decreased, causing reductions in the number of effective panicles, the total number of grains per panicle, the sink capacity per tiller, and grain yield. In rice transplanted at 30- and 35-day seedling ages, increasing cultivation density significantly enhanced the number of effective panicles and grain yield. Additionally, there existed strong, positive correlations between sink capacity per tiller and pre-anthesis dry matter remobilization efficiency and pre-anthesis dry matter contribution to grain yield. We conclude that in addition to cultivation density, enhancing the amount of pre-anthesis dry matter and the remobilization efficiency could be feasible for mitigating grain yield loss caused by delayed transplantation.

## Introduction

With the increasing scarcity of the rural labour force, mechanical transplantation has become a prevalent and simplified cultivation method to replace hand transplantation in Chinese rice production. Many researchers have demonstrated that the achievement of high grain yield for mechanically transplanted rice requires an optimal seedling age^1^. Shen *et al*. reported that among all agronomic practices influencing grain yield of mechanically transplanted rice, seedling age is the most important factor followed by the use of fertilizer and sowing density^2^. Currently, the delay of transplantation events frequently occurs in wheat-rice rotation districts in China. These delays are attributed to the late harvest of the former crop, the deficiency in the number of transplanting machines, and unfavourable weather conditions^3–5^. The seedling age of mechanically transplanted rice even exceeds 30 days in some regions. Today, some regions are facing the challenges of grain yield fluctuations resulting from delayed mechanical transplantation^3,5,6^. Based on previous research and local rice production, the suitable seedling age for mechanically transplanted rice is commonly considered to be less than 25 days^3^.

When the seedling age is more than 25 days, rice seedlings are usually named “delayed transplanted rice” or “rice with old seedling age.” Delaying transplantation can detrimentally impact rice growth and grain yield formation, mainly due to poor tiller occurrence, shortened vegetative duration, and decreased dry matter accumulation^6^. Accordingly, how to effectively compensate for the grain yield loss caused by delaying transplantation has become an important issue for some researchers. Lampayan *et al*. found that lowering the seeding rate could improve the grain yield of rice with old seedling age^7^. Yu *et al*. suggested that for mechanically transplanted rice, adopting dry seedling-nursery technology was instrumental in enhancing seedling quality and prolonging seedling age suitable for transplantation^8^. Hu *et al*. reported that an optimum nitrogen application pattern (a split application ration of 4:3:3 before transplantation, at tillering and at panicle initiation stages) could significantly enhance the nitrogen utilization efficiency and grain yield of delayed mechanically transplanted rice^9^. These reports fully confirmed that it could be feasible to mitigate the negative influence induced by delayed transplantation through various agronomic measurements.

It has been established that planting density plays an important role in improving population structure, promoting the efficient use of sunlight and regulating rice tiller occurrence and grain yield formation^10–12^. Wang *et al*. found that the maximum grain yield was produced at 25 cm × 17 cm spacing for mechanical hill direct sown rice^13^. Qian *et al*. reported that the achievement of high grain yield for mechanically transplanted rice cultivars with small, medium, and large panicles required 4 seedlings per hill, 3 seedlings per hill, and 2 seedlings per hill, respectively^14^. Xu *et al*. showed that an increase in the mechanical transplanting density could significantly enhance the grain yield of rice cultivars suitable for close-planting^15^. Furthermore, it has been verified that the number of effective panicles significantly reduced for rice undergoing delayed transplantation, due to the repression of tiller occurrence, thereby leading to decreased grain yield^6^. Accordingly, increasing the tiller number may be a feasible method for offsetting the grain yield loss of delayed transplanted rice. Whether enhancing cultivation density (i.e., seedling number per hill) can adequately promote the number of effective tillers in rice populations and eventually decrease yield loss under delayed transplantation conditions remains unexplored. However, in regards to optimal seedling age and old seedling age, there is little information available on how cultivation density impacts the utilization efficiency of preanthesis stored photoassimilates and post-anthesis newly created assimilates. Conducting related research could provide a reference for guiding the management of mechanically transplanted rice cultivation.

The objectives of this research were (1) to determine the effect of cultivation density on tiller occurrence, pre-anthesis dry matter remobilization and utilization efficiencies, post-anthesis photosynthetic products use efficiency, sink capacity per tiller, and the grain yield component of rice with optimal and old seedling ages; (2) to ascertain whether increasing the cultivation density could significantly compensate for the grain yield loss of mechanically transplanted rice incurred by delaying transplantation; and (3) to clarify the relationship between the utilization efficiency of photoassimilates and sink capacity.

## Results

### Performances of the main growth duration and tiller occurrence in rice with different seedling ages at normal and high densities

**Figure 1:**
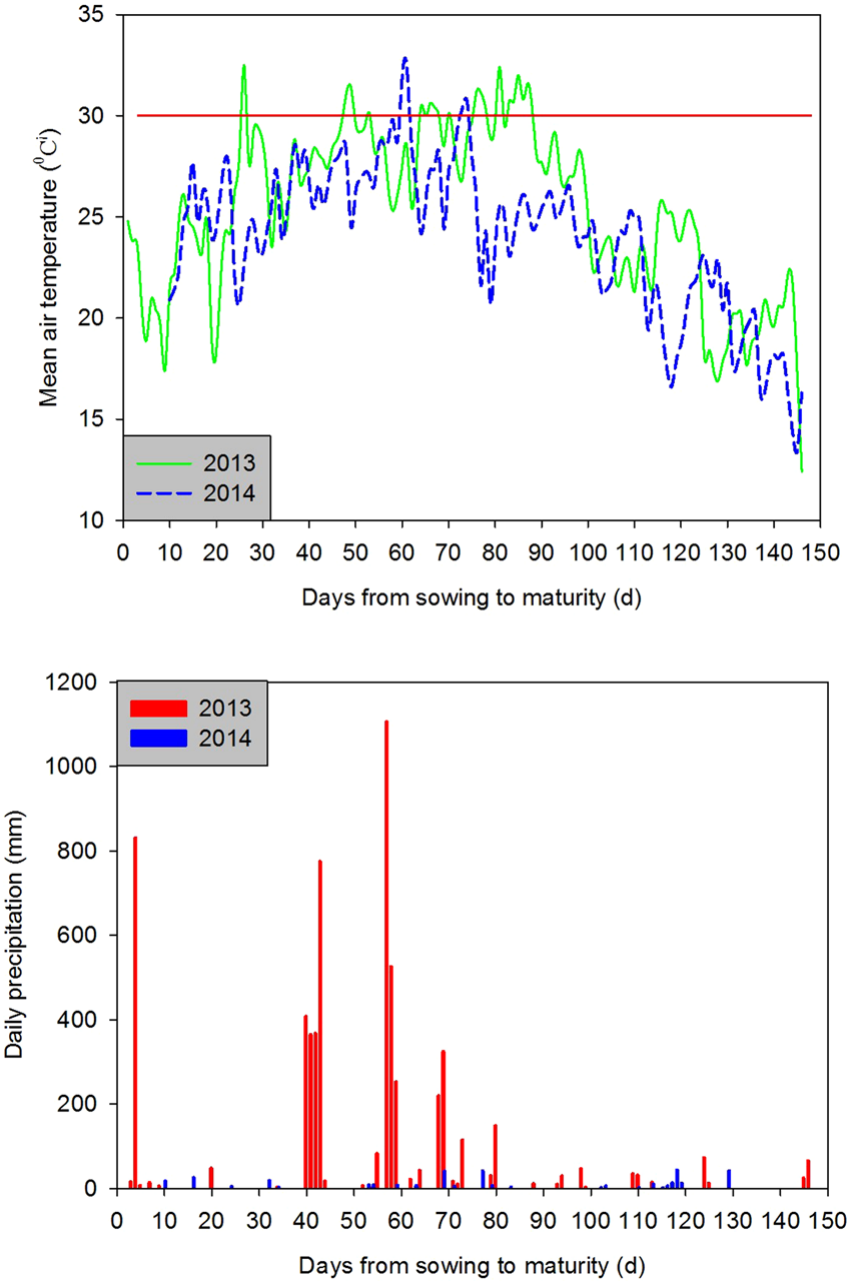
Daily precipitation and mean air temperature from sowing to maturity.

**Figure 2:**
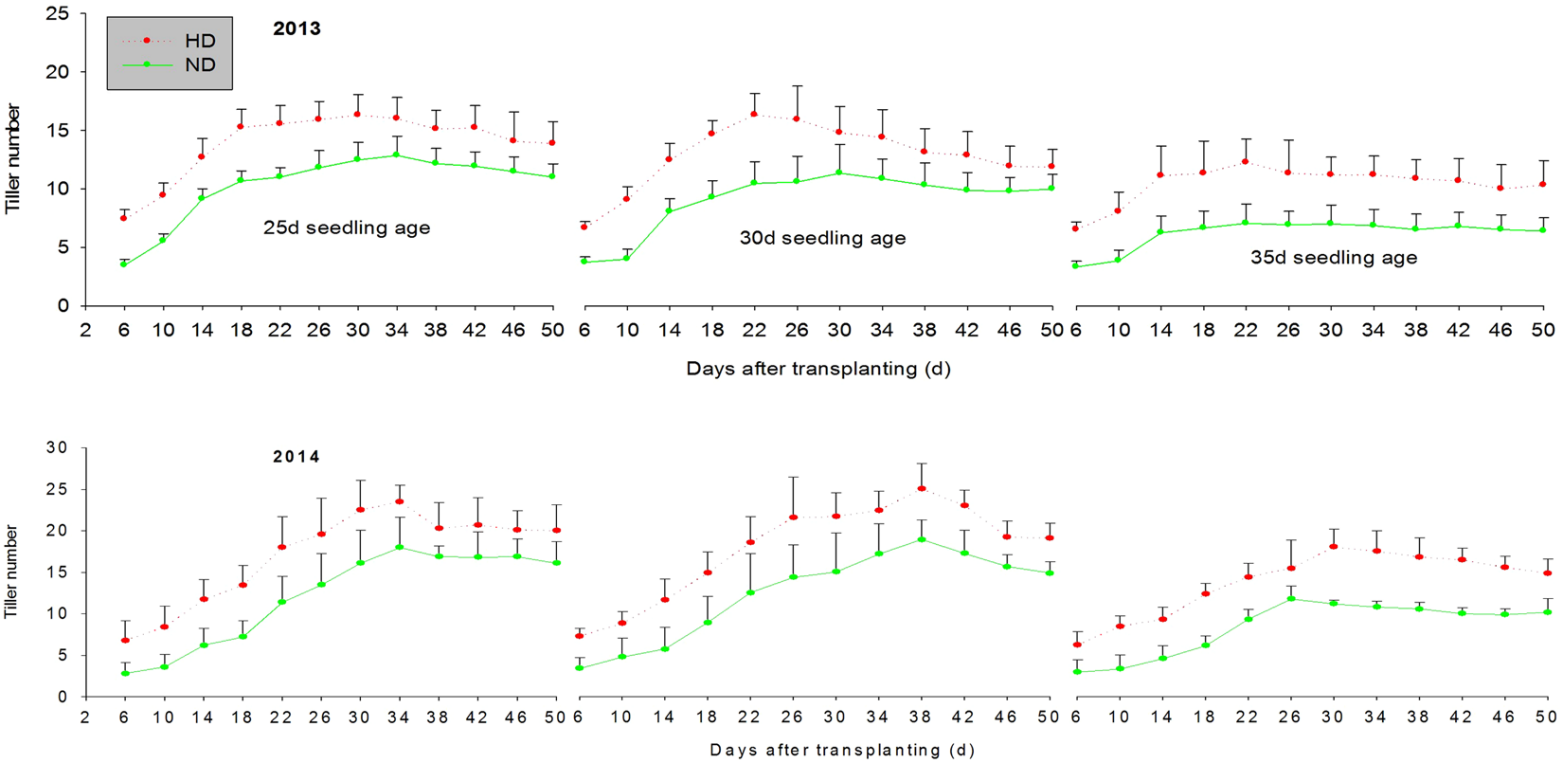
Tiller number of rice with different seedling age at normal and high cultivation densities from the 6th d to the 50th d after transplanting. (**a**) ND = normal density. (**b**) HD = high density.

With the increase of seedling age, the booting, heading, and maturity stages of rice were markedly postponed. However, these parameters remained unchanged at normal and high densities for rice with different seedling ages, indicating that cultivation density did not have an influence on the duration of rice growth (Table 1). In 2013, the booting, heading, and maturity stages of rice with 30- and 35-day seedling ages were delayed by 3–6 days, 2–3 days, and 1–3 days, respectively, when compared with those of rice with a 25-day seedling age. In 2014, these stages were delayed by 3–5 days, 2 days and 1–2 days, respectively. From the 6^th^ day to the 50^th^ day after transplanting, the tiller numbers of rice at high cultivation density were much greater than those at normal cultivation density in both study years, regardless of rice seedling age (Fig. 1). For rice with a 25-day seedling age, the tiller numbers at high cultivation density were 25.94% and 29.04% higher than those at normal cultivation density in 2013 and 2014, respectively (Fig. 2). For rice with a 30-day seedling age, the tiller numbers at high cultivation density were 29.70% and 30.36% higher than those at normal cultivation density in 2013 and 2014, respectively. Finally, for rice with a 35-day seedling age, the tiller numbers at high cultivation density were 40.55% and 39.07% higher than those at normal cultivation density in 2013 and 2014, respectively. The difference in tiller numbers between normal and high densities became greater with the increase in seedling age.

**Table 1:** Dates for booting, heading and maturity stages of rice with different seedling ages at normal and high densities.

Year	Treatment		Dates for different growth stage		
2013	Seedling age	Cultivation density	Booting	Heading	Maturity
	25 d	ND	21-Jul	16-Aug	11-Oct
		HD	21-Jul	16-Aug	11-Oct
	30 d	ND	24-Jul	17-Aug	13-Oct
		HD	24-Jul	17-Aug	13-Oct
	35 d	ND	27-Jul	19-Aug	14-Oct
		HD	27-Jul	19-Aug	14-Oct
2014	25 d	ND	23-Jul	20-Aug	13-Oct
		HD	23-Jul	20-Aug	13-Oct
	30 d	ND	25-Jul	20-Aug	14-Oct
		HD	25-Jul	20-Aug	14-Oct
	35 d	ND	28-Jul	22-Aug	15-Oct
		HD	28-Jul	22-Aug	15-Oct

ND = normal density, HD = high density.

### Dry matter productivity and utilization characteristics in rice with different seedling ages at normal and high densities

At the heading stage, the total dry matter weight, as well as weights of the leaf, stem and sheath, and panicle, at normal density were greater than those at high density in both study years, irrespective of seedling age (Table 2). The parameters mentioned above showed similar tendencies (except for the leaf dry matter weight) at each rice maturity stage in both years. In 2013 and 2014, the dry matter weights of the leaf, stem and sheath, and panicle, and the total weight at heading stage for rice with 30- and 35-day seedling ages showed a decreasing trend compared to that for rice with a 25-day seedling age. Similar results (excluding the dry matter weight of the leaf) were also observed at the maturity stage.

**Table 2:** Above-ground dry matter weight per tiller for rice with different seedling age at normal and high densities

Year	Treatment		Dry matter weight at heading stage (g tiller^−1^)				Dry matter weight at maturity stage (g tiller^−1^)			
2013	Seedling age	Cultivation density	Leaf	Stem and sheath	Panicle	Total	Leaf	Stem and sheath	Panicle	Total
	25 d	ND	0.808a	1.595a	0.532a	2.935a	0.356a	1.530a	2.034a	3.920a
		HD	0.755b	1.450b	0.493a	2.698b	0.392a	1.340b	1.950b	3.682b
	30 d	ND	0.783a	1.385a	0.436a	2.604a	0.461a	1.372a	1.740a	3.573a
		HD	0.741a	1.258b	0.419a	2.418b	0.465a	1.216b	1.702a	3.383b
	35 d	ND	0.731a	1.212a	0.393a	2.336a	0.451a	1.210a	1.574a	3.235a
		HD	0.683b	1.130b	0.384a	2.197a	0.435a	1.125a	1.498b	3.058b
	Seedling age 25d		0.782a	1.523a	0.513a	2.817a	0.374a	1.435a	1.992a	3.801a
	Seedling age 30d		0.762a	1.322b	0.428a	2.511b	0.463a	1.294b	1.721b	3.478b
	Seedling age 35d		0.707b	1.171c	0.389b	2.267c	0.443a	1.168c	1.536c	3.147c
	Normal density		0.774a	1.397a	0.454a	2.625a	0.423a	1.371a	1.783a	3.576a
	High density		0.726a	1.279b	0.432a	2.438b	0.431a	1.227b	1.717b	3.374b
2014	25 d	ND	0.821a	1.612a	0.578a	3.011a	0.368a	1.545a	2.205a	4.118a
		HD	0.788b	1.570a	0.523a	2.881b	0.412a	1.551a	2.016b	3.979b
	30 d	ND	0.801a	1.525a	0.501a	2.827a	0.428a	1.491a	1.981a	3.900a
		HD	0.774a	1.474b	0.483a	2.731a	0.412a	1.420a	1.968a	3.800b
	35 d	ND	0.751a	1.424a	0.435a	2.610a	0.461a	1.400a	1.701a	3.562a
		HD	0.725a	1.382a	0.400a	2.507b	0.472a	1.300b	1.685a	3.457b
	Seedling age 25d		0.805a	1.591a	0.551a	2.946a	0.390a	1.548a	2.111a	4.049a
	Seedling age 30d		0.788a	1.500b	0.492a	2.779b	0.420a	1.456a	1.975a	3.850b
	Seedling age 35d		0.738b	1.403c	0.418b	2.559c	0.467a	1.350b	1.693b	3.510c
	Normal density		0.791a	1.520a	0.505a	2.816a	0.419a	1.479a	1.962a	3.860a
	High density		0.762a	1.475a	0.469a	2.706b	0.432a	1.424a	1.890b	3.745b

Values followed by different letters represent significant difference at *p* < 0.05. ND = normal density, HD = high density. ND = normal density, HD = high density.

Irrespective of seedling age, the leaf dry matter remobilization amount, remobilization efficiency, contribution to grain yield, and post-anthesis photosynthesis amount decreased while the stem and sheath dry matter remobilization amount, remobilization efficiency, contribution to grain yield, and post-anthesis photosynthesis contribution to grain yield were enhanced at high density, compared with those at normal density. In 2013, the corresponding values for leaf were 15.67%, 10.31%, 12.94%, and 1.47%, respectively. They were 11.29%, 7.87%, 8.90%, and 0.48%, respectively, in 2014. With increasing seedling age, the post-anthesis photosynthesis contribution to grain yield decreased while the other parameters increased across the two study years (Table 3).

**Table 3:** Pre-anthesis dry matter remobilization traits and post-anthesis newly created photosynthesis in rice with different seedling age at normal and high densities

Year	Treatment		Leaf			Stem and sheath				Post-anthesis photosynthesis contribution to grain yield (%)
	Seedling age	Cultivation density	Dry matter remobilization amount (g)	Dry matter remobilization efficiency (%)	Dry matter contribution to grain yield (%)	Dry matter remobilization amount (g)	Dry matter remobilization efficiency (%)	Dry matter contribution to grain yield (%)	Post-anthesis photosynthesis amount (g)	
2013	25 d	ND	0.452a	55.94a	30.09a	0.065a	4.08a	4.33a	0.985a	65.58a
		HD	0.363b	48.08b	24.91b	0.110a	7.59b	7.55b	0.984a	67.54b
	30 d	ND	0.322a	41.12a	24.69a	0.013a	0.94a	1.00a	0.969a	74.31a
		HD	0.276b	37.25b	21.51b	0.042a	3.34b	3.27a	0.965b	75.21a
	35 d	ND	0.280a	38.30a	23.71a	0.002a	0.17a	0.17a	0.899a	76.12a
		HD	0.248b	36.31a	22.26a	0.005a	0.44b	0.45b	0.861b	77.29a
	Seedling age 25d		0.408a	52.14a	27.54a	0.087a	5.75a	5.91a	0.985a	66.54a
	Seedling age 30d		0.299b	39.24b	23.12b	0.027b	2.08b	2.13b	0.967a	74.76b
	Seedling age 35d		0.264c	37.34b	23.01b	0.003c	0.30c	0.31c	0.880b	76.69c
	Normal density		0.351a	45.39a	26.44a	0.027a	1.91a	2.01a	0.951a	71.56a
	High density		0.296b	40.71b	23.02b	0.052a	4.09b	4.07b	0.937b	72.91a
2014	25 d	ND	0.453a	55.18a	27.84a	0.067a	4.16a	4.12a	1.107a	68.04a
		HD	0.376b	47.72b	25.18b	0.019b	1.21b	1.27b	1.098b	73.54b
	30 d	ND	0.373a	46.57a	25.20a	0.034a	2.24a	2.30a	1.073a	72.49a
		HD	0.362a	46.77a	24.38a	0.054a	3.66a	3.64a	1.069a	71.99a
	35 d	ND	0.290a	38.62a	22.91a	0.024a	1.69a	1.90a	0.952a	75.20a
		HD	0.253a	34.90b	19.69b	0.082b	5.93b	6.38b	0.950a	73.93a
	Seedling age 25d		0.415a	51.52a	26.57a	0.043a	2.70a	2.76a	1.103a	70.67a
	Seedling age 30d		0.368b	46.67b	24.79b	0.044a	2.94a	2.97a	1.071b	72.24b
	Seedling age 35d		0.272c	36.79c	21.29c	0.053a	3.78a	4.16a	0.951b	74.56c
	Normal density		0.372a	47.03a	25.52a	0.042a	2.74a	2.86a	1.044a	71.62a
	High density		0.330a	43.33b	23.25b	0.052a	3.50b	3.64b	1.039a	73.12a

Values followed by different letters represent significant difference at *p* < 0.05. ND = normal density, HD = high density.

### Sink capacity per tiller and the grain yield component of rice with different seedling ages at normal and high densities

**Figure 3:**
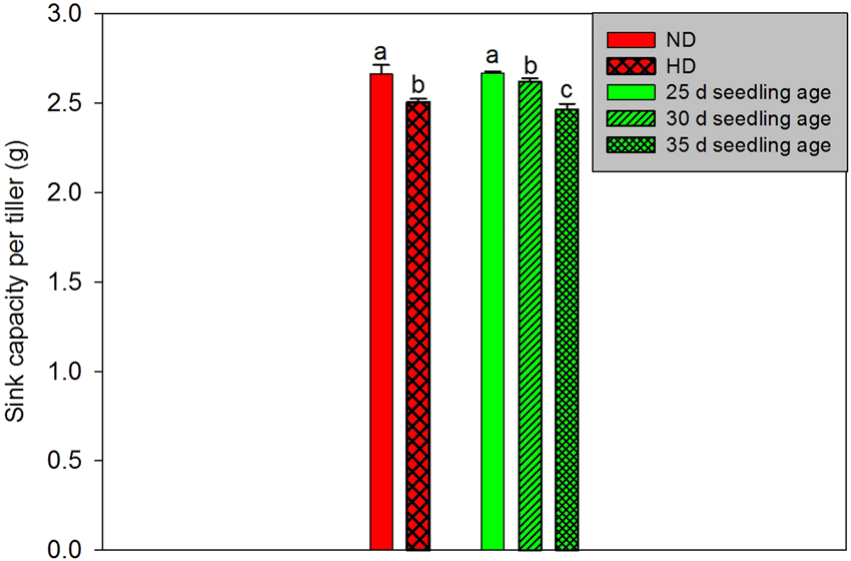
Effects of seedling age and cultivation density on sink capacity per tiller of rice. (**a**) Values followed by different letters represent significant difference at *p < *0.05. (**b**) ND = normal density. (**c**) HD = high density.

As shown in Fig. 3, compared to that at normal density, sink capacity per tiller decreased at high density. Sink capacity per tiller decreased with increasing seedling age. The change tendencies of the parameters of grain yield components were similar in both study years (Table 4). The numbers of primary and secondary branches per panicle and the total number of grains per panicle at high density were found to be 6.14%, 7.47%, and 6.21% lower, respectively, than those at normal density in 2013. In 2014, the corresponding values were 9.38%, 10.07%, and 7.03%, respectively. Conversely, the number of effective panicles per square metre and grain yield at high density were increased by 17.55% and 6.73% in 2013 and 14.67% and 6.82% in 2014, respectively, compared with those at normal density. As seedling age increased, the numbers of the secondary branches per panicle, the total grains per panicle, and the numbers of the effective panicles per square metre decreased across both study years. For rice with a 25-day seedling age, there was no significant difference in grain yield between normal density and high density across both study years. However, in regards to rice with 30- and 35-day seedling ages, the grain yields at high density were enhanced by 6.63% and 13.10% in 2013 and 7.67% and 12.02% in 2014, respectively, when compared to that at normal density.

**Table 4:** Agronomic traits and grain yield of rice with different seedling age at normal and high cultivation density.

Year	Treatment		No. of the primary branch per panicle	No. of the secondary branch per panicle	No. of effective panicles per square meters	No. of total grains per panicle	Seed-setting rate (%)	1000-grain weight (g)	Grain yield (kg ha^−1^)
	Seedling age	Cultivation density							
2013	25 d	ND	9.88a	19.43a	415.50a	100.06a	83.85a	24.69a	7290.89a
		HD	9.83a	18.01a	453.65b	94.27b	82.19a	24.79a	7482.67a
	30 d	ND	10.22a	16.28a	384.34a	97.06a	81.88a	24.67a	6451.82a
		HD	9.22b	15.56a	443.83b	91.06b	81.76a	24.89a	6879.83b
	35 d	ND	10.35a	14.50a	249.99a	96.29a	80.44a	24.77a	4802.41a
		HD	9.53a	12.91a	336.60b	89.85b	80.70a	24.99a	5431.43b
	Seedling age 25d		9.86a	18.72a	434.58a	97.17a	82.02a	24.74a	7386.78a
	Seedling age 30d		9.72a	15.92b	414.08b	94.06a	81.82a	24.78a	6665.83b
	Seedling age 35d		9.94a	13.71c	293.29c	93.07a	80.57b	24.88a	5116.92c
	Normal density		10.15a	16.74a	349.94a	97.80a	82.06a	24.71a	6181.71a
	High density		9.53a	15.49a	411.36b	91.73b	80.88a	24.89a	6597.98b
2014	25 d	ND	12.50a	25.20a	448.74a	112.61a	95.10a	24.79a	9434.71a
		HD	12.00a	24.60a	490.76b	106.30b	96.02a	24.33a	9694.85a
	30 d	ND	12.20a	24.90a	427.66a	110.50a	95.49a	24.83a	8874.44a
		HD	11.30a	20.90b	483.06b	103.00b	94.44a	25.16a	9554.78b
	35 d	ND	9.75a	12.75a	304.70a	101.00a	94.97a	24.40a	5712.86a
		HD	7.92b	11.02b	380.50b	92.04b	95.25a	25.09a	6399.66b
	Seedling age 25d		12.25a	24.90a	469.75a	109.46a	95.56a	24.56a	9564.78a
	Seedling age 30d		11.75a	22.90b	455.36b	106.75a	94.97a	25.00a	9214.61b
	Seedling age 35d		8.84b	11.89c	342.60c	96.52b	95.11a	24.75a	6056.26c
	Normal density		11.48a	20.95a	393.70a	108.04a	95.19a	24.67a	8004.00a
	High density		10.41b	18.84b	451.44b	100.45b	95.24a	24.86a	8549.76b

Values followed by different letters represent significant difference at *p* < 0.05. ND = normal density, HD = high density.

### Relationships between sink capacity and pre-anthesis dry matter remobilization and post-anthesis photosynthesis utilization characteristics

**Figure 4:**
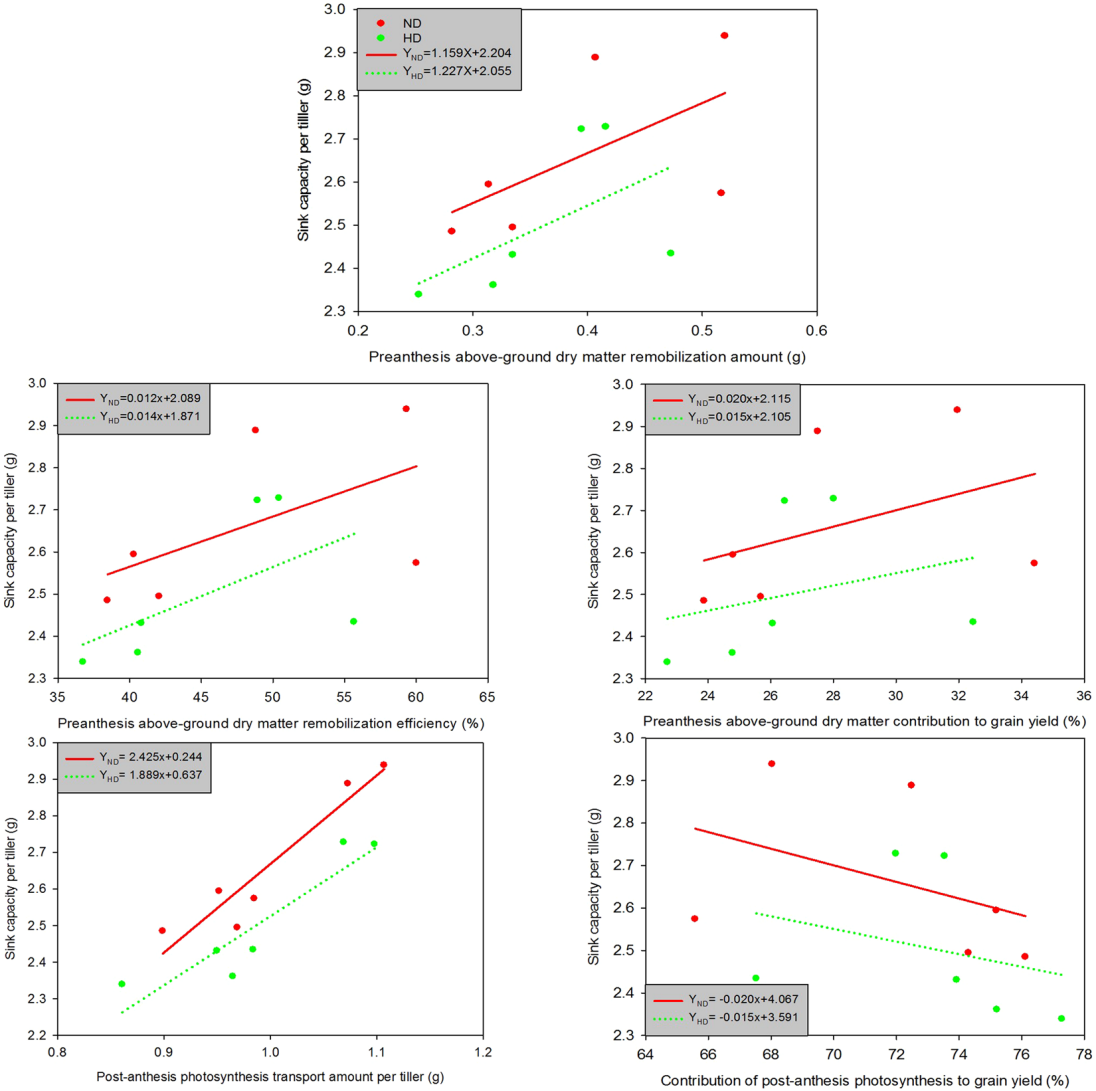
Relationships between sink capacity per tiller and utilization efficiency of pre- and post-anthesis photosynthesis in rice under high and normal densities conditions. (**a**)ND = normal density. (**b**)HD = high density.

The relationships between sink capacity per tiller and the utilization efficiency of pre-anthesis dry matter and post-anthesis photosynthesis under high and normal densities are shown in Fig. 4. There were positive relationships between sink capacity per tiller and pre-anthesis dry matter remobilization amount, remobilization efficiency, contribution to grain yield and the amount of post-anthesis photosynthesis transport per tiller. Conversely, sink capacity per tiller was negatively related to the contribution of post-anthesis photosynthesis to grain yield.

**Figure 5:**
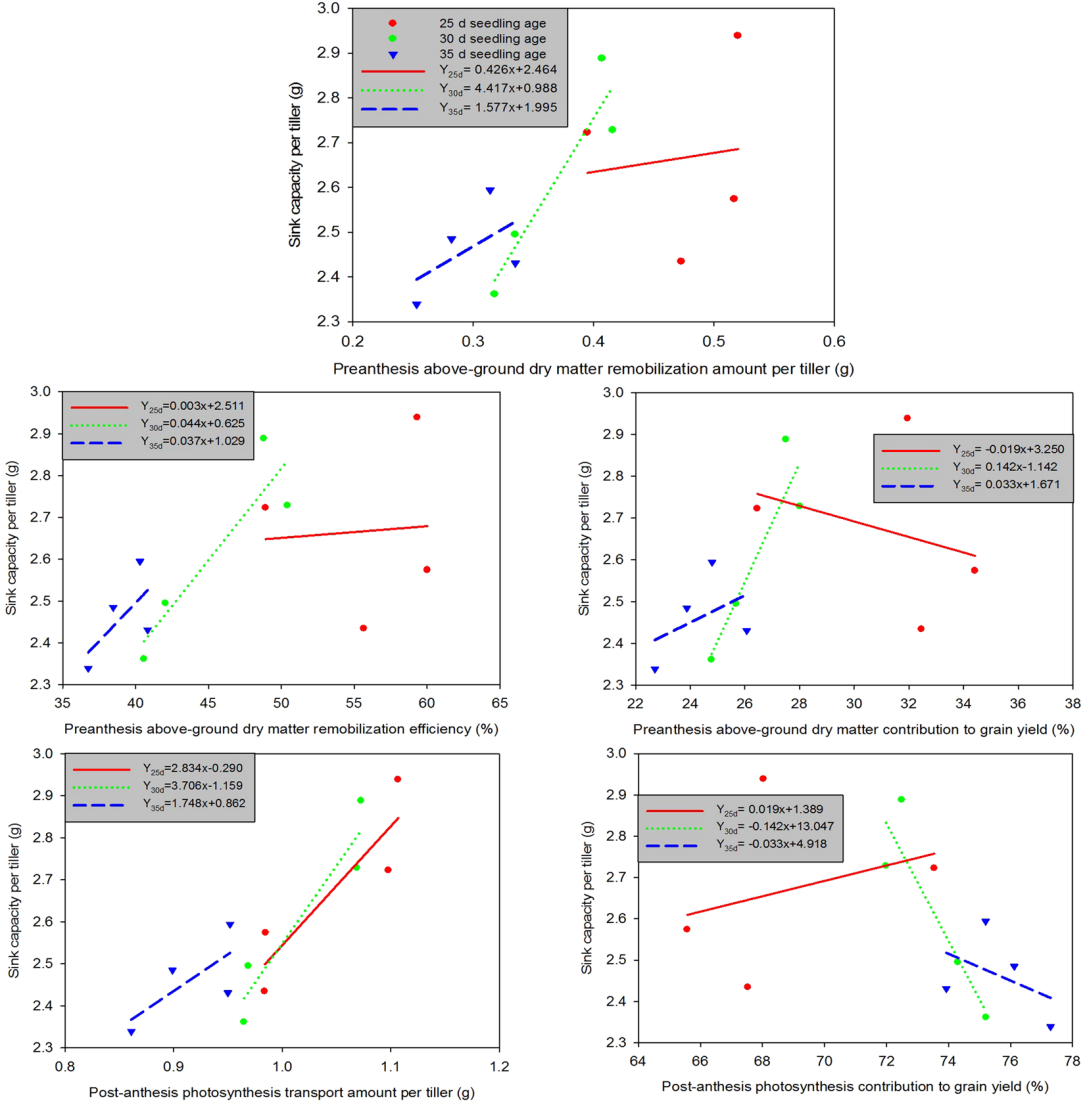
Relationships between sink capacity per tiller and utilization efficiency of pre-anthesis dry matter and post-anthesis photosynthesis in rice with different seedling ages.

Across all seedling ages, sink capacity per tiller was positively correlated with the amounts of pre-anthesis dry matter remobilization and post-anthesis photosynthesis transport per tiller (Fig. 5). For rice with a 25-day seedling age, sink capacity per tiller was negatively related to the preanthesis dry matter contribution to grain yield and positively related to the post-anthesis photosynthesis contribution to grain yield. In rice with 30- and 35-day seedling ages, sink capacity per tiller was positively related to the pre-anthesis dry matter contribution to grain yield and negatively related to the post-anthesis photosynthesis contribution to grain yield.

## Discussion

### Effects of cultivation densities on growth duration and tiller occurrence of mechanically transplanted rice with different seedling ages

Previous reports showed that rice jointing, heading, and maturity stages under mechanical transplantation were delayed compared with those under hand transplantation, with this difference increasing with the delay of mechanical transplantation^6^. In the present study, rice booting, heading, and maturity stages were delayed by 1–6 days with increasing seedling age. The rice booting stage was delayed more than the other growth stages. Compared to that of rice with a 25-day seedling age, the booting stage was delayed only by 2–3 days for rice with a 30-day seedling age and 5–6 days for rice with a 35-day seedling age, suggesting that the rice vegetative stage is obviously shortened with the delay of mechanical transplantation. In contrast, cultivation density did not show any influence on rice growth stage regardless of seedling age. This phenomenon indicates that seedling age could be an important factor determining rice growth stage under mechanical transplantation patterns, which is in agreement with past research^7,16^.

It is indisputable that rice tiller occurrence is depressed with increasing seedling age, thereby resulting in reduced tiller numbers^16–19^. In general, the seedling nursery of mechanically transplanted rice is characterized by a greater sowing amount. With the prolongation of transplantation, the growth of rice seedlings is evidently suppressed due to intensive competition among individual plants under limited growing space in seedling nursery beds, which further hampers rice tiller occurrence after being transplanted to the paddy field^16,20^. In the present study, rice tiller number markedly decreased with an increase in seedling age regardless of density, suggesting that rice seedling age could play a vital role in determining tiller occurrence. In addition, cultivation density is also an important factor influencing rice tiller number. Increasing the seedling number per hill or the distance among rows has been shown to be feasible in enhancing the tiller numbers of the rice population^21,22^. Data in our study also showed an increase in tiller number through enhanced cultivation density regardless of seedling age. However, it is notable that, as for rice with a 25-day seedling age, the increased magnitude of the average tiller number induced by increasing cultivation density was smaller than that of rice with 30- and 35-day seedling ages. These results reveal that an enhancement in cultivation density exerts a positive role in minimizing the reduction of rice tiller number incurred by delaying transplantation.

### Effects of cultivation densities on the utilization efficiency of pre-anthesis dry matter stored in vegetal organs and post-anthesis photoassimilates of mechanically transplanted rice with different seedling ages

For rice that experienced delayed transplantation, we observed the amount of above-ground dry matter accumulation after transplantation substantially decreased due to inhibited photosynthetic ability compared with rice transplanted on time^6,19^. We also observed that the above-ground dry matter weight of mechanically transplanted rice from heading to maturity decreased with an increase in seedling age. Furthermore, our study further verified that the amount of pre-anthesis dry matter remobilization, efficiency and contribution to grain yield, as well as the amount of post-anthesis photosynthesis, were reduced significantly with increasing rice seedling age. One possible reason for this phenomenon is the shortened vegetative growth of delayed mechanically transplanted rice, which not only adversely influences pre-anthesis dry matter accumulation but also constrains post-anthesis photosynthesis^1,6^. In addition, previous work showed that the remobilization efficiency and contribution to grain yield of dry matter stored in the leaf, stem and sheath before the rice heading stage reduced significantly with increasing seedling numbers per hill^15^. Our results indicated that increasing cultivation density significantly reduced the amount of pre-anthesis dry matter remobilization, efficiency and contribution to grain yield, and the amount of post-anthesis photosynthesis. With increasing seedling age and cultivation density, both the amounts of pre-anthesis dry matter remobilization and post-anthesis photosynthesis decreased. Nonetheless, it is notable that while the pre-anthesis dry matter contribution to grain yield decreased, the post-anthesis photosynthesis contribution to grain yield increased. We infer that the amount of pre-anthesis dry matter remobilization decreased more than that of post-anthesis photosynthesis, thereby resulting in a reduced pre-anthesis dry matter contribution to grain yield and an increased post-anthesis photosynthesis contribution to grain yield.

### Effects of cultivation densities on grain yield and yield components of mechanically transplanted rice with different seedling ages

Previous studies demonstrated that delaying transplantation triggered a decrease in rice grain yield due to a marked reduction in the number of effective panicles and grain number per panicle^1,6,7^. In the present study, we observed that with the enhancement of seedling age, rice grain yield and sink capacity per tiller pronouncedly declined because of the significant decreases in the number of effective panicles per square metre and the number of grains per panicle. This could be attributed to inhibited tiller occurrence and shortened vegetative growth for delayed transplanted rice^1,6,17^. It is notable that increasing cultivation density significantly enhanced grain yield for rice with 30- and 35-day seedling ages, but it showed a nonsignificant influence on that of rice with a 25-day seedling age. Despite a reduced grain number per panicle, the increase of cultivation density produced more effective panicles, eventually enhancing the grain yield of rice with 30- and 35-day seedling ages. This result suggests that the increase in cultivation density could effectively compensate for rice grain yield loss resulting from delayed transplantation. Additionally, we found that with increasing seedling age or cultivation density, the significant reduction in grain number per panicle was primarily attributable to the marked decrease in the number of secondary branches per panicle (Table 4).

### Agronomic channels for compensating for the grain yield loss of mechanically transplanted rice resulting from delaying transplantation

Considering the detrimental effect on rice grain yield due to delaying transplantation, researchers have made many attempts to improve the grain yield of mechanically transplanted rice^1,9,23^. Hu *et al*. and Liu *et al*. confirmed that a moderate increase in nitrogen top-dressing at the rice panicle initiation stage could mitigate the adverse effect and offset partial grain yield losses caused by delaying transplantation^9,23^. Since the significant reduction in grain yield was primarily attributed to the decrease in the number of effective panicles for delayed transplanted rice, enhancing cultivation density might minimize the decrease in magnitude of the number of effective panicles and compensate for grain yield loss. However, this still lacks evidence because related reports are limited, especially for mechanically transplanted rice. Our study confirmed that for rice that experienced delayed transplantation, increasing the cultivation density (enhancing the seedling number per hill from 3 to 6) significantly enhanced the number of effective panicles despite a marginal decrease in the total number of grains per panicle, thereby compensating for grain yield loss. This result indicates that a rational enhancement of cultivation density could effectively expand a rice population and improve grain yield, particularly for rice plants that received inhibiting effects because of delayed transplantation.

Apart from the number of effective panicles, sink capacity per tiller (determined by the number of total grains per panicle and one thousand grain weight) is also an important factor determining rice grain yield^24,25^. Our study indicated that the increase of cultivation density enhanced the number of effective panicles but decreased sink capacity per tiller. Therefore, increasing sink capacity per tiller is essential for further improvement of grain yield with delayed transplantation. As a rule, the carbohydrate supply for grain filling mainly relies on two resources: (1) dry matter stored in vegetative organs before heading stage, and (2) newly created photosynthesis from heading to maturity stage, during which the contribution of the former to final grain yield accounts for approximately 30%^26–30^. Currently, increasing evidence has demonstrated that non-structural carbohydrates, the main source of the dry matter stored in vegetative organs, not only function as an assimilate supply for grain filling but also play a critical role in enhancing sink activity during rice grain filling^31,32^. This fully embodies the importance of dry matter accumulated in vegetative organs before heading for determining sink capacity and final grain yield^33,34^. In the present study, we further found that in regard to mechanical-transplanted rice that experienced delayed transplantation, enhancing the amount of pre-anthesis dry matter remobilization and the contribution to grain yield was an effective channel to increase sink capacity per tiller regardless of cultivation density. As a result, agronomic measurements such as irrigation and fertilizer managements should be utilized to promote pre-anthesis dry matter accumulation and remobilization. Related research still needs further exploration and will be of great significance for guiding rice production.

## Materials and Methods

### Plant materials and experimental design

The field experiments were carried out at Yutai (35°00′N, 116°39′E), Shandong, China, during rice growth stages (from May to October) in 2013 and 2014. The site belongs to typical wheat-rice rotation cropping district in China. The daily average temperature and precipitation during rice growth season of both years were shown in Fig. 1. The means of daily average temperature in 2013 was 1.28 °C higher than that in 2014, particularly the means from the 83th day to the 100th day after sowing. The precipitation in 2013 was 42.01 mm, which was 39.46 mm higher than that in 2014. A locally cultivated *japonica* rice cultivar Shengdao19, which had been bred by Shandong Rice Research Institute, was employed as experimental material. The experiment was arranged as a completely randomized split-plot design with three replications. Rice plants with three differed seedling ages (representing the days from sowing to transplantation) were assigned as the main plot, with two cultivation densities being randomly allotted to sub-plot. Three seedling ages were 25d, 30d and 35d, respectively. Planting 3 seedlings per hill and 6 seedlings per hill were regarded as two different cultivation densities (normal density and high density). To keep independent irrigation and fertilization managements for rice plants with three seedling ages, the main plots were separated by 50-cm-wide ridges with plastic film inserted into soil at a depth of 30 cm.

### Cultivation management

For all treatments, rice seeds were uniformly sown on 23 May under mechanical transplantation pattern. Rice plants were transplanted on 17 June, 22 June and 27 June, respectively, by a transplanter (KUBATO, SPU-60) made in Japan. The distances between rows and hills were 30 cm and 12 cm, respectively, with 3 seedlings per hill and 6 seedlings per hill as normal cultivation density and high cultivation density. The size of each sub-plot was 30 m^2^. Fertilization and other agronomic managements were implemented according to local rice cultivation practices. The total N application amount was 270 kg ha^−1^ with an N, P and K application rate of 2:1:1.

### Sampling and determination

The tiller numbers of 20 fixed hills from each plot were counted from the 6^th^ day to the 50^th^ day after transplantation at an internal of 3 days. Rice growth stages of each plot were recorded timely. Representative plants of 6 hills from each plot were sampled at rice heading and maturity stages. The above-ground parts of these plants were oven-dried at 105 °C for 0.5 h and divided into three segments, including leaf blades, stems and sheaths and panicles. Subsequently, these samples were oven-dried at 80 °C until constant weight and weighed to acquire dry matter weights, respectively. At maturity, rice plants of 10 m^2^ from each plot were harvested excluding border plants to determine grain yield with moisture content 14%. Thirty Representative hills of rice plants from each plot were sampled to determine the number of effective panicles, total and filled grains per panicle and 1000-grain weight.

### Data analysis

Based on the parameters mentioned above, the amount of pre-anthesis dry matter remobilization and efficiency, the dry matter contribution to grain yield, the amount of post-anthesis photosynthesis, the post-anthesis photosynthesis contribution to grain yield, and sink capacity per tiller were calculated using the following equations^31,35,36^:

Pre-anthesis dry matter remobilization amount (g) = Dry matter weight of the leaf or stem and sheath per tiller at heading - dry matter weight of the leaf or stem and sheath per tiller at maturity.

Pre-anthesis dry matter remobilization efficiency (%) = 100 × dry matter remobilization amount/dry matter weight per tiller at heading.

Pre-anthesis dry matter contribution to grain yield (%) = 100 × dry matter remobilization amount/(dry weight of the grains per tiller at maturity - dry weight of the grains per tiller at heading).

Post-anthesis photosynthesis amount (g) = Dry matter weight of the above - ground per tiller at maturity - Dry matter weight of the above-ground per tiller at heading.

Post-anthesis photosynthesis contribution to grain yield (%) = (Dry matter weight of the above -ground per tiller at maturity − Dry matter weight of the above-ground per tiller at heading)/(dry weight of the grains per tiller at maturity − dry weight of the grains per tiller at heading).

Sink capacity per tiller = (Number of total grains per panicle × one thousand grain weight)/1000.
